# Immune‐Checkpoint‐Inhibitor‐Related Bullous Pemphigoid During Avelumab Maintenance Therapy in a Patient With Metastatic Renal Pelvic Carcinoma: A Case Report

**DOI:** 10.1002/iju5.70240

**Published:** 2026-09-01

**Authors:** Taketoshi Nara, Kengo Furihata, Akane Kikuchi, Yuya Sekine, Hiromi Sato, Mizuki Kobayashi, Ryohei Yamamoto, Mitsuru Saito, Shintaro Narita, Tomonori Habuchi

**Affiliations:** ^1^ Department of Urology Japanese Red Cross Akita Hospital Akita Japan; ^2^ Department of Urology Akita University Graduate School of Medicine Akita Japan; ^3^ Department of Urology Japanese Red Cross Osaka Hospital Osaka Japan

**Keywords:** avelumab, bullous pemphigoid, immune checkpoint inhibitors, urothelial carcinoma

## Abstract

**Introduction:**

Bullous pemphigoid is a rare cutaneous, immune‐related adverse event, and avelumab‐associated cases are extremely rare.

**Case Presentation:**

A 76‐year‐old man receiving avelumab maintenance therapy for recurrent renal pelvic carcinoma developed pruritic erythema and tense blisters 20 days after the first dose. Laboratory examination showed eosinophilia, increased immunoglobulin E level, and markedly elevated anti‐BP180 antibodies. Moreover, skin biopsy revealed subepidermal blistering with eosinophilic infiltration, supporting drug‐associated bullous pemphigoid. Prednisolone (15 mg/day, increased to 25 mg/day) led to marked improvement. After the second course, avelumab was discontinued, and no recurrence occurred.

**Conclusion:**

Immune‐checkpoint‐inhibitor‐related, drug‐associated bullous pemphigoid can present with nonspecific symptoms before blistering. Thus, urologists should monitor skin changes during immune checkpoint inhibitor therapy and promptly involve dermatology.

## Keynote Message

Considering that avelumab‐associated bullous pemphigoid may initially present with nonspecific cutaneous symptoms, early dermatologic evaluation is important for diagnosis and management.

## 1 Introduction

Avelumab (an anti‐programmed death‐ligand 1 [PD‐L1] antibody) is a standard maintenance therapy for advanced urothelial carcinoma after platinum‐based chemotherapy [1]. During immune checkpoint inhibitor (ICI) therapy, cutaneous immune‐related adverse events (irAEs) are common. However, bullous pemphigoid (BP) is rare, with cases associated with avelumab being extremely rare [2, 3, 4]. Herein, we report an ICI‐related, drug‐associated BP (DABP) during avelumab maintenance therapy for recurrent renal pelvic carcinoma.

## 2 Case Presentation

A 76‐year‐old man with right renal pelvic carcinoma (cT3N0M0) underwent right laparoscopic nephroureterectomy and partial cystectomy 8 years earlier (pathology: invasive urothelial carcinoma, pT3pN0). After postoperative administration of gemcitabine plus cisplatin, pulmonary metastases emerged 1 year later, which progressed after multiple systemic therapies. After two courses of dose‐dense methotrexate, vinblastine, doxorubicin, and cisplatin, avelumab maintenance therapy was initiated, resulting in stable disease.

However, pruritic erythema and blisters appeared 20 days after the first dose (Figure 1). Dermatology was consulted, and the patient was started on topical corticosteroids and antiallergic agents, leading to temporary improvement. Nevertheless, erythema and blistering worsened after administering the second course on day 30.

**FIGURE 1:**
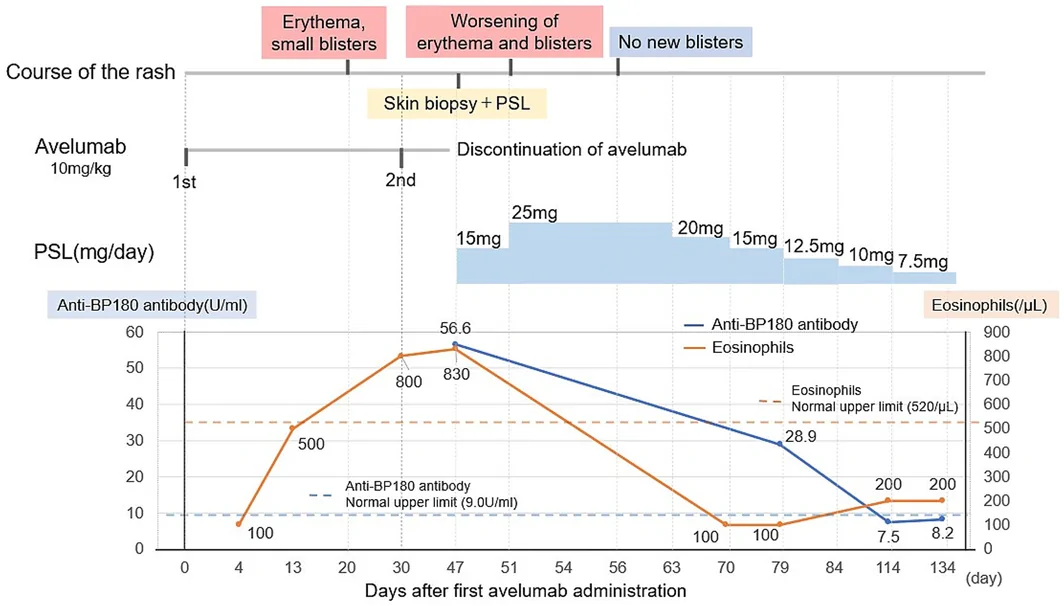
Clinical course. Timeline after the first avelumab administration (day 0). Day 20: Pruritic erythema and blister formation. Day 30: Skin regions worsening after the second avelumab course despite topical corticosteroids. Day 47: Elevated anti‐BP180 antibody, skin biopsy confirming bullous pemphigoid, prednisolone (PSL) initiated at 15 mg/day. Day 51: PSL increased to 25 mg/day due to worsening; gradual improvement ensued. Avelumab was discontinued after the second course.

On day 47, erythema and tense blisters involved both axillae, anterior chest, occiput, and medial thighs (15% body surface area; Figure 2A,B), without mucosal lesions. Laboratory tests revealed eosinophilia (830/μL) and elevated immunoglobulin E (720 IU/mL) and anti‐BP180 antibody levels (56.6 U/mL). Skin biopsy showed subepidermal blistering with predominantly eosinophilic infiltration and mild perivascular inflammation (Figure 3). Based on the clinical course, serology, and histopathology, BP was diagnosed.

**FIGURE 2:**
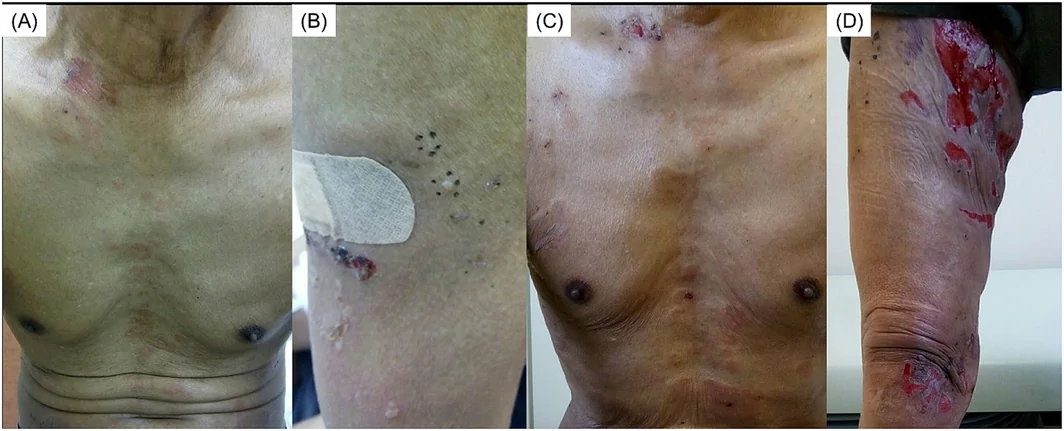
Cutaneous findings. Day 47: (A) pruritic erythema on the anterior chest; (B) small blisters on the left upper extremity. Day 51: (C) extension of erythema over the trunk; (D) worsening erythema with erosions on the right thigh.

**FIGURE 3:**
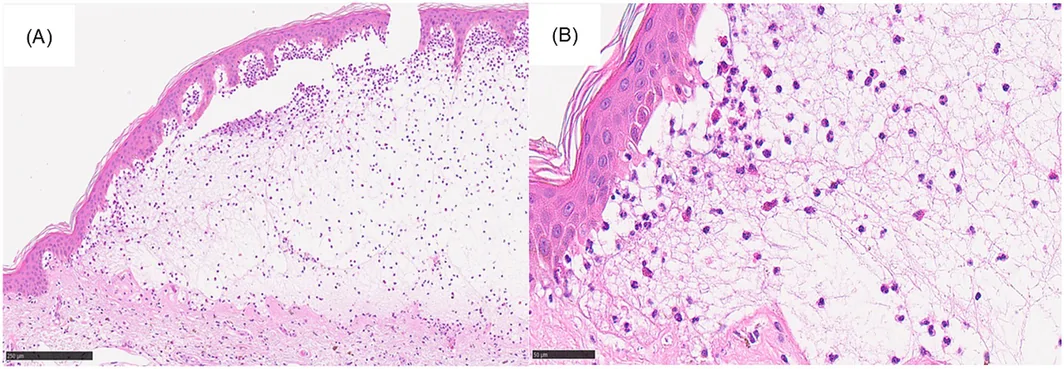
Histopathological findings. Skin biopsy (day 47, left upper arm). Low‐magnification view revealed subepidermal blister formation (A), and high‐magnification examination demonstrated predominantly eosinophilic inflammatory infiltration (B) (hematoxylin–eosin staining).

Prednisolone (PSL, 15 mg/day) was initiated on day 47. However, edematous erythema spread (Figure 2C,D). Thus, PSL was increased to 25 mg/day on day 51. After the skin lesions resolved without new blisters and the anti‐BP180 titer decreased, PSL was tapered. Avelumab was discontinued after the second course, and no skin recurrence occurred. As the underlying malignancy progressed, the patient's general condition worsened, precluding further systemic therapy. The patient died 5 months after avelumab initiation.

## 3 Discussion

ICI‐related DABP developed during avelumab maintenance therapy, with cutaneous symptoms appearing on day 20 (earlier than the reported median onset of 22–26 weeks [3, 4]). Although the reason behind this early onset remains unclear, previous systemic therapies may have altered the patient's immune status and contributed to early onset. Nevertheless, this explanation remains speculative [3, 5, 6]. Avelumab‐associated DABP is extremely rare. In a pooled analysis of 373 ICI‐related DABP cases, avelumab accounted for only one case (0.27%), with most cases being attributed to nivolumab or pembrolizumab [4]. Whether this reflects a true mechanistic difference between programmed death‐1 (PD‐1) and PD‐L1 blockade or clinical exposure differences remains unclear. Further case accumulation is needed.

Conventional BP predominantly affects elderly individuals and presents with pruritic erythema and tense blisters caused by autoantibodies against hemidesmosomal proteins (e.g., BP180 and BP230) [2, 7, 8]. ICI‐related DABP may arise from the disruption of peripheral immune tolerance. PD‐1/PD‐L1 blockade may activate autoreactive T and B cells, driving autoantibody production against these proteins [5, 9, 10]. Furthermore, BP180 expression on tumor cell surfaces may trigger cross‐reactive immunity at the dermal–epidermal junction [5, 6, 11].

Early ICI‐related DABP presents with nonspecific pruritus and erythema before blistering, making differentiation from eczema or drug eruption difficult [2, 3, 4]. Diagnostic evaluation includes skin biopsy and direct immunofluorescence showing linear IgG/C3 deposition at the dermal–epidermal junction [2, 3, 4]. One limitation of this report is that direct immunofluorescence was not performed. Nevertheless, the diagnosis was based on the clinical course, elevated anti‐BP180 antibody level, and histopathology [3, 7, 8]. Anti‐BP180 and anti‐BP230 antibodies are useful for monitoring disease activity [2].

Compared with common cutaneous irAEs, ICI‐related DABP presents distinct clinical and therapeutic features. Common cutaneous irAEs are often diagnosed clinically and managed with topical corticosteroids, systemic corticosteroids, steroid‐sparing agents, or immunotherapy interruption based on their severity [2, 12]. By contrast, ICI‐related DABP may mimic eczema or drug eruption; however, tense blistering, eosinophilia, anti‐BP180/BP230 antibodies, and histopathology are important diagnostic clues [2, 3]. Because DABP treatment generally follows conventional BP strategies, early dermatologic consultation is desirable before empiric escalation to high‐dose systemic corticosteroids [7, 8, 13]. Moreover, ICI‐related DABP may follow a prolonged or relapsing course, complicating ICI continuation or rechallenge [3, 4, 14].

Although ICI‐related DABP treatment follows conventional BP guidelines, systemic corticosteroids may attenuate antitumor immunity [3, 8]. Apalla et al. proposed a stepwise approach wherein mild‐to‐moderate cases are managed with topical and systemic corticosteroids while continuing ICIs, whereas severe or refractory cases warrant ICI interruption and intensified immunosuppression [13]. In the present case, PSL (15 mg/day) was started with dermatology and increased to 25 mg/day before resolution.

Currently, there is no consensus regarding ICI rechallenge after ICI‐associated DABP. According to research, bullous eruptions recurred in 50% of patients after rechallenge [4, 14], although some cases were managed with corticosteroid adjustment while continuing ICIs [9]. Therefore, rechallenge should be individualized based on cutaneous severity, treatment response, and disease activity. In the present case, ICI therapy was discontinued because of cancer progression, and rechallenge was not pursued. Although corticosteroid treatment for irAEs may adversely affect survival outcomes after ICI therapy [15], its effect in this patient remains unclear given his refractory urothelial carcinoma before avelumab initiation.

Therefore, urologists must remain vigilant for cutaneous irAEs with the increasing administration of ICIs in urologic oncology. Early collaboration with dermatology permits timely diagnosis and appropriate management while preserving oncologic treatment when possible.

## 4 Conclusion

We report a rare case of avelumab‐associated DABP presenting with early, nonspecific cutaneous symptoms. Urologists should monitor for skin changes during ICI therapy and promptly involve dermatology to achieve accurate diagnosis and therapeutic interventions.

## Funding

The authors have nothing to report.

## Consent

Written consent to publish this case and accompanying images was obtained from the patient.

## Conflicts of Interest

The authors declare no conflicts of interest.

## Data Availability

Data sharing not applicable to this article as no datasets were generated or analysed during the current study.
